# The Reporting of Cytomorphological Spectrum of Thyroid Lesions Using the Recent Bethesda System: A Study on the Population of Northwest Uttar Pradesh

**DOI:** 10.7759/cureus.114037

**Published:** 2026-08-05

**Authors:** Sucheta Yadav, Vaishali Singh, Medha Misra, Abhilash Abhilash, Neha Singh, Vishakha Gupta

**Affiliations:** 1 Department of Pathology, Government Medical College, Badaun, IND; 2 Department of Immunohematology and Blood Transfusion, Government Medical College, Badaun, IND; 3 Department of Pathology, Dr Bhimrao Ramji Ambedkar Government Medical College, Kannauj, IND; 4 Department of Orthopaedics, Government Medical College, Badaun, IND; 5 Department of Forensic Medicine and Toxicology, Government Medical College, Badaun, IND; 6 Department of Otorhinolaryngology, Government Medical College, Badaun, IND

**Keywords:** bethesda category, cytopathology, fine-needle aspiration, follicular cells, thyroid swellings

## Abstract

Background: Cytomorphological study of fine-needle aspirates has gained wide acceptance as one of the first-line assessments done for thyroid swellings. The Bethesda System for Reporting Thyroid Cytology (TBSRTC) has provided a standard, formal, and effective method of communication between pathologists and clinicians.

Aim: This study aimed to evaluate the cytomorphological spectrum of thyroid lesions using the revised (2023) TBSRTC and to assess cytohistological correlation in cases with available surgical follow-up.

Methods: Patients with thyroid swellings underwent fine-needle aspiration (FNA). Smears were prepared, fixed, and stained with hematoxylin and eosin and May-Grunwald-Giemsa. They were studied under the microscope and categorized according to the revised edition of TBSRTC. Correlation with histopathological diagnosis was analyzed in 54 cases. McNemar’s test was applied to assess diagnostic agreement between cytology and histopathology. The chi-square test and Fisher’s exact test were used to analyze cyto-histological concordance. A p-value <0.05 was considered statistically significant.

Results: The frequency of occurrence of thyroid swellings was approximately sixfold higher in female cases than in male cases. The highest incidence of these swellings was between 21 and 30 years of age. Bethesda category II had the highest prevalence, 292 (82.26%) cases. The cytological and histopathological correlation in 54 cases reported that p-values for category-wise comparisons were highly significant (p < 0.001), except for Bethesda III and IV categories.

Conclusions: FNAC plays an important role in categorizing patients who can be managed by medical intervention alone and those who require surgery. Every category has an associated risk of being diagnosed as a malignancy on histopathological examination. This helps in patient stratification and in determining the extent of surgical management and follow-up required.

## Introduction

Scandinavian investigators Martin and Ellis introduced fine-needle aspiration (FNA) of thyroid swellings as a small-needle aspiration biopsy in the 1960s; this technique was subsequently widely adopted in North America by the 1980s [1,2]. Over the past four decades, FNA has become a widely accepted first-line diagnostic modality for thyroid swellings worldwide. It is an uncomplicated, comparatively safer, economical, and painless technique with a short turnaround time. According to a study by Amrikachi and his co-researchers, fine-needle aspiration cytology (FNAC) had a specificity of 96% and sensitivity of 93% when performed on an adequate sample [3]. Clinically, patients may present with a solitary thyroid nodule or diffuse enlargement of the thyroid gland. During examination, the size, texture, nodularity, tenderness, and movement on deglutition are assessed. A thorough history is the first step in ruling out neoplastic lesions. Development of hoarseness of voice, dysphagia, rapid increase in size of the swelling, history of irradiation, any first-degree relative diagnosed with thyroid malignancy, or presence of hard and irregular nodules points to a neoplasm rather than an inflammatory lesion of the thyroid. Various diagnostic modalities, such as ultrasonography, radionuclide scanning, biochemical evaluation, and FNA, are used to assess thyroid swelling.

A conference on thyroid FNAC was organized by the National Cancer Institute (NCI) at Bethesda, MD, in October 2007. Thereafter, the use of evidence-based standardized terminology for reporting thyroid FNAC was recommended [4]. The Bethesda System for Reporting Thyroid Cytology (TBSRTC) was introduced for seamless communication among clinicians and pathologists.

Its first edition was published in 2010 and was modified in 2017. The third edition of TBSRTC was published in 2023. The TBSRTC is based on six diagnostic categories; each category has an implied risk of malignancy (ROM) and a recommendation for clinical management [5,6]. The recent edition of TBSRTC focuses on aligning the Bethesda nomenclature with the 2022 World Health Organization (WHO) Classification of Thyroid Neoplasms. TBSRTC 2023 provides each category with an implied cancer risk. Based on prospectively analyzed large series with surgical follow-up reported since the 2017 edition, the ROMs have been updated.

This study was conducted to analyze the cytomorphological features of thyroid lesions in the population of north-west Uttar Pradesh and categorize them according to the revised TBSRTC and categorize them according to the revised TBSRTC, and to correlate the cytological findings with histopathological outcomes in cases undergoing surgical resection.

## Materials and methods

Study design

An observational retrospective study was conducted over 3.5 years (December 2021 to June 2025) in the Department of Pathology at Government Medical College, Badaun.

Study participants

All patients with thyroid swellings referred from the departments of otorhinolaryngology, surgery, and medicine for FNAC were included in the study. Relevant details pertaining to demographics, clinical history, thyroid function tests, and ultrasound imaging were collected before the procedure. Cases with insufficient clinical/demographic data or repeat aspirates performed on the same nodule within the study period were excluded from the analysis. 

Ethical considerations

This study was approved by the Institutional Ethics Committee (IEC No: 21/IEC/GMCB/2025). Written informed consent was obtained from all participants. The study was conducted in accordance with the Declaration of Helsinki of 1975, revised in 2000. 

Procedure

Patients were asked to lie in a recumbent position; a small support was placed under their neck, making the thyroid region prominent; patients were asked to avoid swallowing during the procedure. The gland was palpated, and the skin overlying the swelling was disinfected using an isopropyl alcohol (70%) swab. FNA was performed, maintaining aseptic conditions using a non-aspirate technique. The needle was passed back and forth into the lesion at different angulations. The material was drawn into the needle by capillary action and expelled onto a glass slide using an air-filled syringe. This method was adapted from a technique introduced by A. Zajdela and his co-workers [7]. A disposable needle (22- or 23-gauge) attached to a plastic disposable syringe (20 mL) was used. Four smears were prepared for each case. The smears were fixed by air-drying or in 95% ethanol, depending upon the stain used. This parallel use of air-dried and wet-fixed smears is recommended as the two staining methods are complementary [8]. May-Grunwald-Giemsa (MGG) stain provides good cytoplasmic details, and nuclear details are well-visualized in a wet, fixed hematoxylin and eosin (H&E)-stained smear. Three smears were stained with MGG, and one with H&E. FNA smears were scanned to check for adequacy. Adequacy criteria in FNAC depend on the type of tissue from which the sample is taken. A thyroid FNA sample is considered adequate if it contains six clusters of undistorted, well-stained, and unobstructed follicular cells; the cells should be at least 10 in number [5]. The amount of cellularity, cytomorphological features, and background were carefully examined under a microscope. FNA was performed under ultrasound guidance in all cases. After clinical and radiological correlation, they were reported and categorized using TBSRTC-3rd edition (Table 1). All slides reported before 2023 were retrieved from the archives, examined microscopically, and re-categorized according to the latest edition of TBSRTC (Table 2). Histopathological correlation was done in 54 cases in which thyroid surgery was done. These 54 cases represented all patients within the study cohort who underwent subsequent surgical resection during the study period; no additional selection criteria were applied.

**Table 1:** The Bethesda System for Reporting Thyroid Cytology (TBSRTC) 2023 Adapted from Ref. [5].

Bethesda category	Nomenclature	Different diagnostic entities/Sub-category
Category I	Non-diagnostic (ND)	Cyst fluid only
		Virtually acellular specimen
		Others (obscuring blood, clotting artifact, drying artifact, etc.)
Category II	Benign	Consistent with follicular nodular disease (include adenomatoid nodule, colloid nodule, etc.)
		Consistent with chronic lymphocytic (Hashimoto) thyroiditis in the proper clinical context
		Consistent with granulomatous (subacute) thyroiditis
		Others
Category III	Atypia of undetermined significance (AUS)	AUS -- nuclear atypia
		AUS -- other
Category IV	Follicular neoplasm (FN)	Follicular neoplasm
		Oncocytic (Hurthle cell) follicular neoplasm (OFN)
Category V	Suspicious for malignancy	Suspicious for papillary thyroid carcinoma
		Suspicious for medullary thyroid carcinoma
		Suspicious for metastatic carcinoma
		Suspicious for lymphoma
Category VI	Malignant	Papillary thyroid malignancy
		High-grade follicular-derived carcinoma
		Medullary thyroid malignancy
		Undifferentiated (anaplastic) thyroid carcinoma
		Squamous cell carcinoma
		Carcinoma with mixed features
		Metastatic malignancy
		Non-Hodgkin lymphoma
		Other

**Table 2:** The 2023 Bethesda System for Reporting Thyroid Cytopathology: Implied risk of malignancy with expected ranges based on follow-up of surgically resected nodules with recommended clinical management Adapted from Ref. [5]. FNA, fine-needle aspiration.

Diagnostic category	Risk of malignancy mean % (range)	Usual management
Non-diagnostic	13 (5-20)	Repeat FNA with ultrasound guidance
Benign	4 (2-7)	Clinical and ultrasound follow-up
Atypia of undetermined significance	22 (13-30)	Repeat FNA, molecular testing, diagnostic lobectomy, surveillance
Follicular neoplasm	30 (23-34)	Molecular testing, diagnostic lobectomy
Suspicious of malignancy	74 (67-83)	Molecular testing, lobectomy, or near-total thyroidectomy
Malignant	97 (97-100)	Lobectomy or near-total thyroidectomy

Statistical analysis

All quantitative data obtained were tabulated in Microsoft Excel Version 2021 (Microsoft Corp., Redmond, WA, USA). Statistical analysis was performed using standard statistical software SPSS Version 26.0 (IBM Corp., Armonk, NY, USA). Descriptive statistics were presented as frequencies and percentages. Diagnostic performance metrics, including sensitivity, specificity, positive predictive value (PPV), negative predictive value (NPV), and accuracy, were calculated with 95% confidence intervals (CI). McNemar’s test was applied to assess diagnostic agreement between cytology and histopathology. The chi-square test and Fisher’s exact test were used to analyze cyto-histological concordance. A p-value <0.05 was considered statistically significant. For diagnostic performance analysis, Bethesda categories V and VI were considered cytologically "positive" for malignancy and category II was considered "negative"; categories III and IV (indeterminate) were excluded from this specific calculation, consistent with standard TBSRTC risk-stratified reporting.

## Results

During the entire duration of the study (December 2021 to June 2025), 369 patients with anterior midline neck swellings were referred for FNA, out of which 14 were reported as thyroglossal cysts, which were more common in the younger age group (0-10 years) and were predominantly seen in male patients.

Out of 355 thyroid swellings, 303 were reported in female cases and 52 in male cases; the ratio of presentation in male and female patients was 1:5.8. The gender distribution is presented in Table 3.

**Table 3:** Sex distribution in thyroid lesions

Gender	Total cases (n = 355)	%
Female	303	85.35
Male	52	14.65

Patients ranged in age from one to 70 years; the most common age group was the third decade (21-30 years). The age-wise distribution of thyroid swelling is given in Table 4. All patients presented with a midline anterior neck swelling, barring one who presented with cervical lymphadenopathy without a clinically significant midline thyroid swelling. FNA from a small thyroid nodule was later reported as papillary carcinoma of the thyroid gland.

**Table 4:** Age distribution of thyroid lesions

Age group	Total (n = 355)	%
1-10	6	1.69
11-20	63	17.75
21-30	106	29.86
31-40	78	21.97
41-50	56	15.77
51-60	35	9.86
61-70	11	3.10

All cases were categorized in accordance with the third edition of TBSRTC, as shown in Table 5.

**Table 5:** Distribution of thyroid lesions using Bethesda system

S. no.	Bethesda category	Number of cases according to category (total = 355)	Different diagnostic entities/sub-category	Number of cases according to sub-category (total = 355)
1.	Category I: Non-diagnostic (ND)	30 (8.45%)	Cyst fluid only	8 (2.25%)
			Virtually acellular specimen	19 (5.35%)
			Others (obscuring blood, clotting artifact, etc.)	3 (0.85%)
2.	Category II: Benign	292 (82.26%)	Consistent with follicular nodular disease (includes adenomatoid nodule, colloid nodule, etc.)	246 (69.30%)
			Consistent with chronic lymphocytic (Hashimoto) thyroiditis in the proper clinical context	37 (10.42%)
			Consistent with granulomatous (subacute) thyroiditis	8 (2.25%)
			Others (consistent with acute thyroiditis)	1 (0.28%)
3.	Category III: Atypia of undetermined significance (AUS)	9 (2.54%)	AUS -- nuclear atypia	7 (1.97%)
			AUS -- other	2 (0.56%)
4.	Category IV: Follicular neoplasm (FN)	8 (2.25%)	Follicular neoplasm	7 (1.97%)
			Oncocytic (Hurthle cell) follicular neoplasm (OFN)	1 (0.28%)
5.	Category V: Suspicious for malignancy	8 (2.25%)	Suspicious for papillary thyroid carcinoma	8 (2.25%)
			Suspicious for medullary thyroid carcinoma	0
			Suspicious for metastatic carcinoma	0
			Suspicious for lymphoma	0
6.	Category VI: Malignant	8 (2.25%)	Papillary thyroid malignancy	6 (1.69%)
			High-grade follicular-derived carcinoma	0
			Medullary thyroid malignancy	1 (0.28%)
			Undifferentiated (anaplastic) thyroid carcinoma	1 (0.28%)
			Squamous cell carcinoma	0
			Carcinoma with mixed features	0
			Metastatic malignancy	0
			Non- Hodgkin lymphoma	0
			Other	0

**Figure 1:**
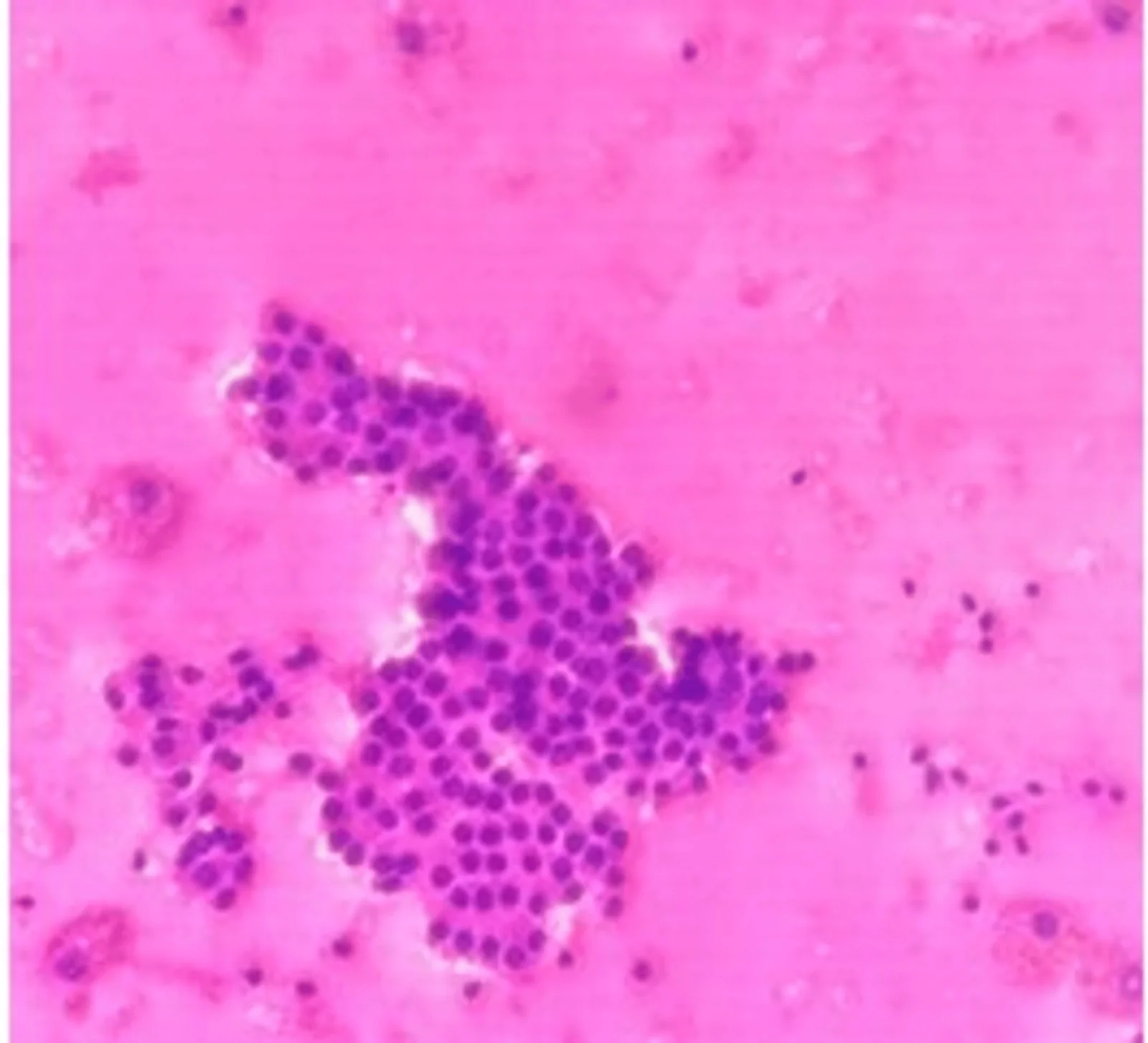
Benign (consistent with follicular nodular disease) follicular cells arranged in tight clusters in a colloid-rich background consistent with colloid nodule (H&E stain, 400× magnification) H&E, hematoxylin and eosin.

Thirty smears were placed in Bethesda category I (non-diagnostic) and subcategorized as paucicellular (19 cases), obscured by blood (three cases), and showing only cystic fluid (eight cases). Bethesda Category II had the largest number of cases, 246 of which were consistent with follicular nodular disease. The smears were moderately cellular, comprising benign follicular cells with round to oval nuclei arranged in sheets and clusters and as single cells (Figure 1).

**Figure 2:**
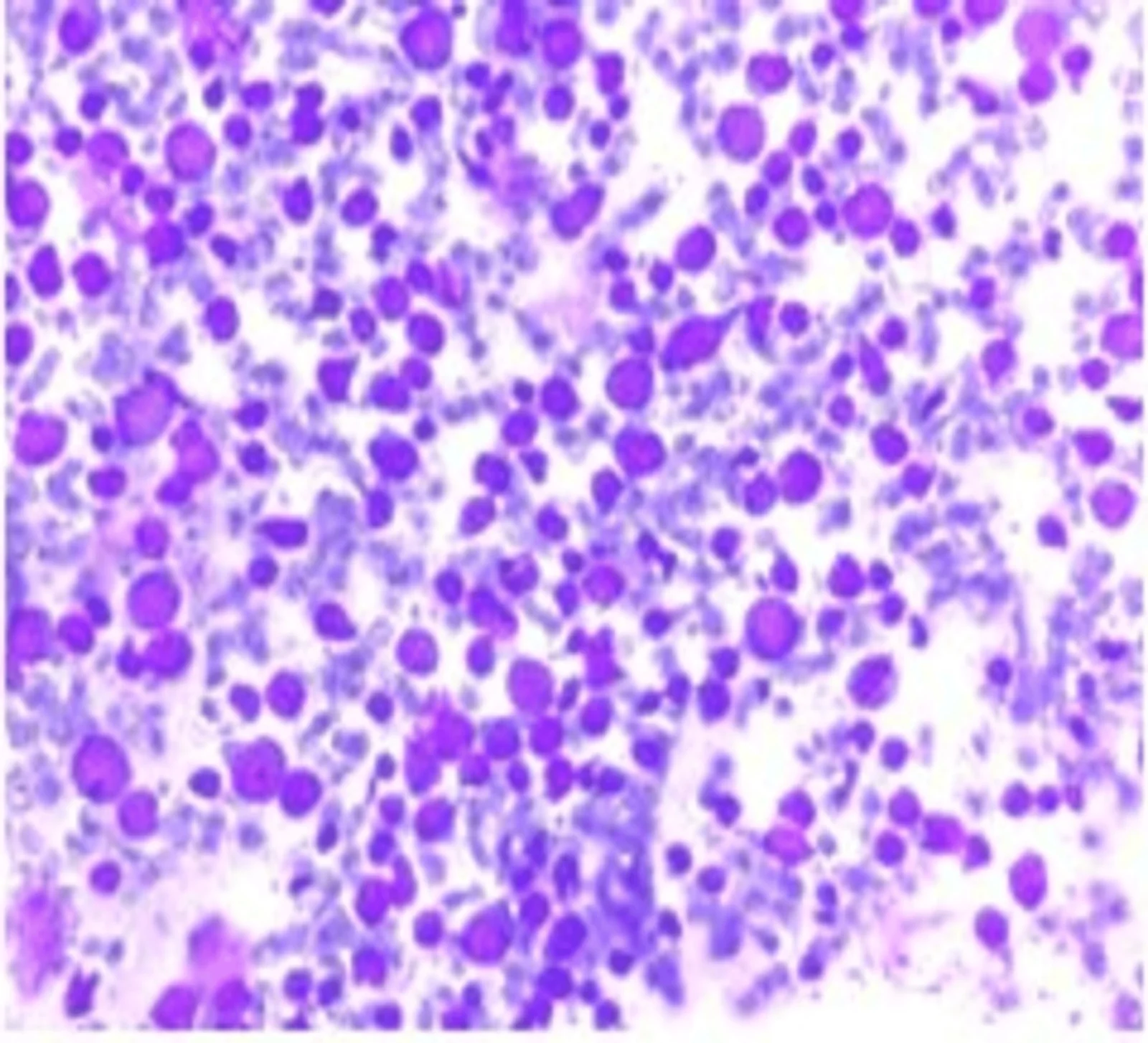
Benign (consistent with chronic lymphocytic (Hashimoto) thyroiditis in the proper clinical context) lymphocytic thyroiditis showing florid inflammation consisting of a polymorphic lymphoid cell population and few follicular cells (MGG stain, 400× magnification) MGG, May-Grunwald-Giemsa.

The cells were present in a colloid-mixed hemorrhagic background. Mild anisonucleosis and focal degeneration admixed with cystic macrophages were also seen in 137 cases (56%) of follicular nodular disease. In the subcategory consistent with lymphocytic thyroiditis, smears showed follicular epithelial cells present in clusters as well as single cells with an impinging lymphoid cell population (Figure 2).

Degenerative changes in follicular epithelial cells were also noted. The background was hemorrhagic and occasionally showed the presence of plasma cells, polymorphs, lymphohistiocytic aggregates, epithelioid cells, occasional Hurthle cells, and binucleated cells.

**Figure 3:**
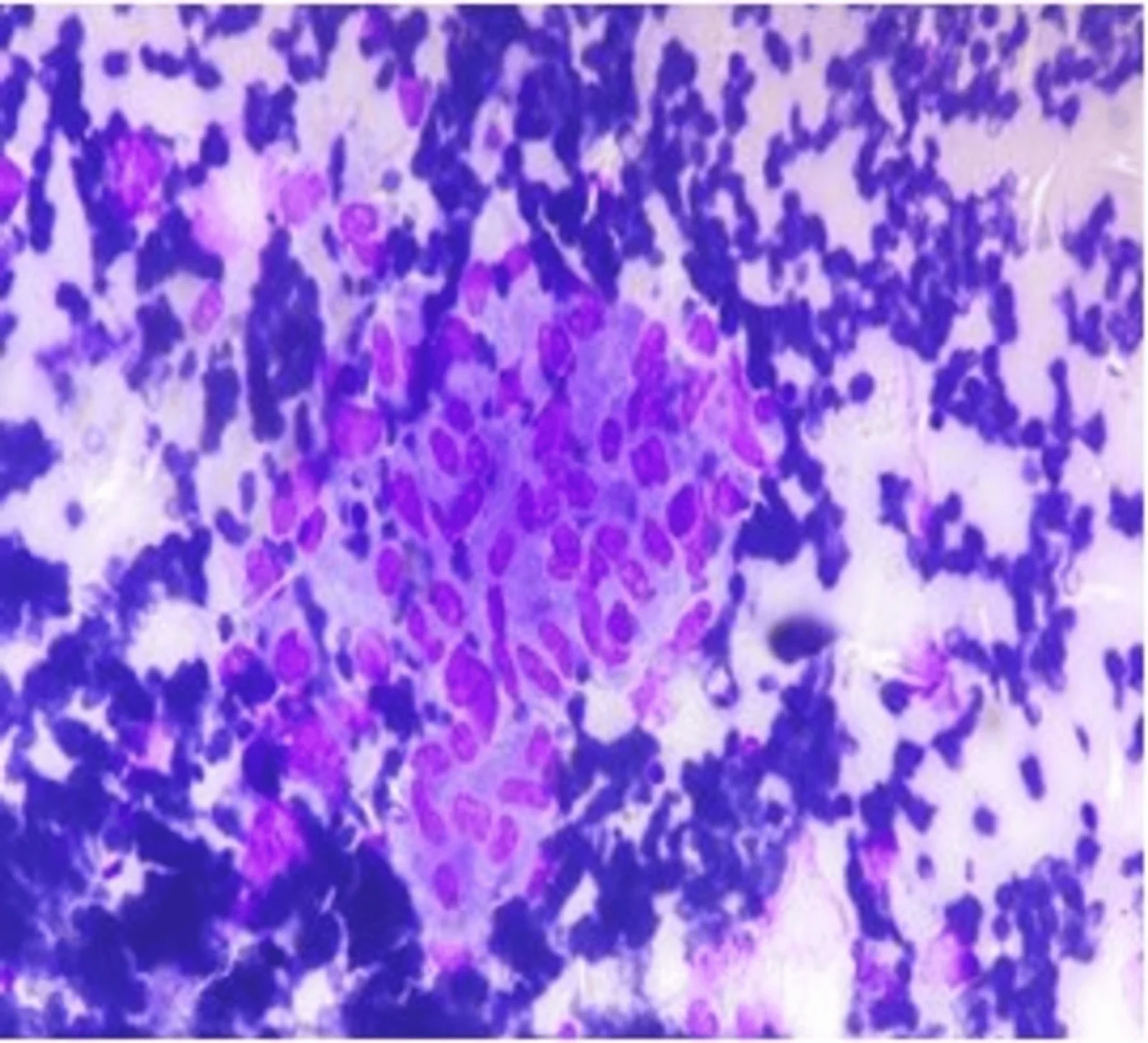
Benign (consistent with granulomatous (subacute) thyroiditis) granulomatous (subacute) thyroiditis, showing benign follicular epithelial cells admixed with epithelioid histiocytes (MGG stain, 400× magnification) MGG, May-Grunwald-Giemsa.

Smears consistent with granulomatous (subacute) thyroiditis showed benign follicular cells present individually, in sheets, and in clusters. Few clusters of tissue macrophages (histiocytes) and occasional multinucleated giant cells were seen in a colloid-mixed hemorrhagic background. One case was consistent with acute thyroiditis; the smear showed abundant inflammatory cell infiltrate composed of polymorphs and lymphocytes, with a few scattered benign follicular cells (Figure 3).

**Figure 4:**
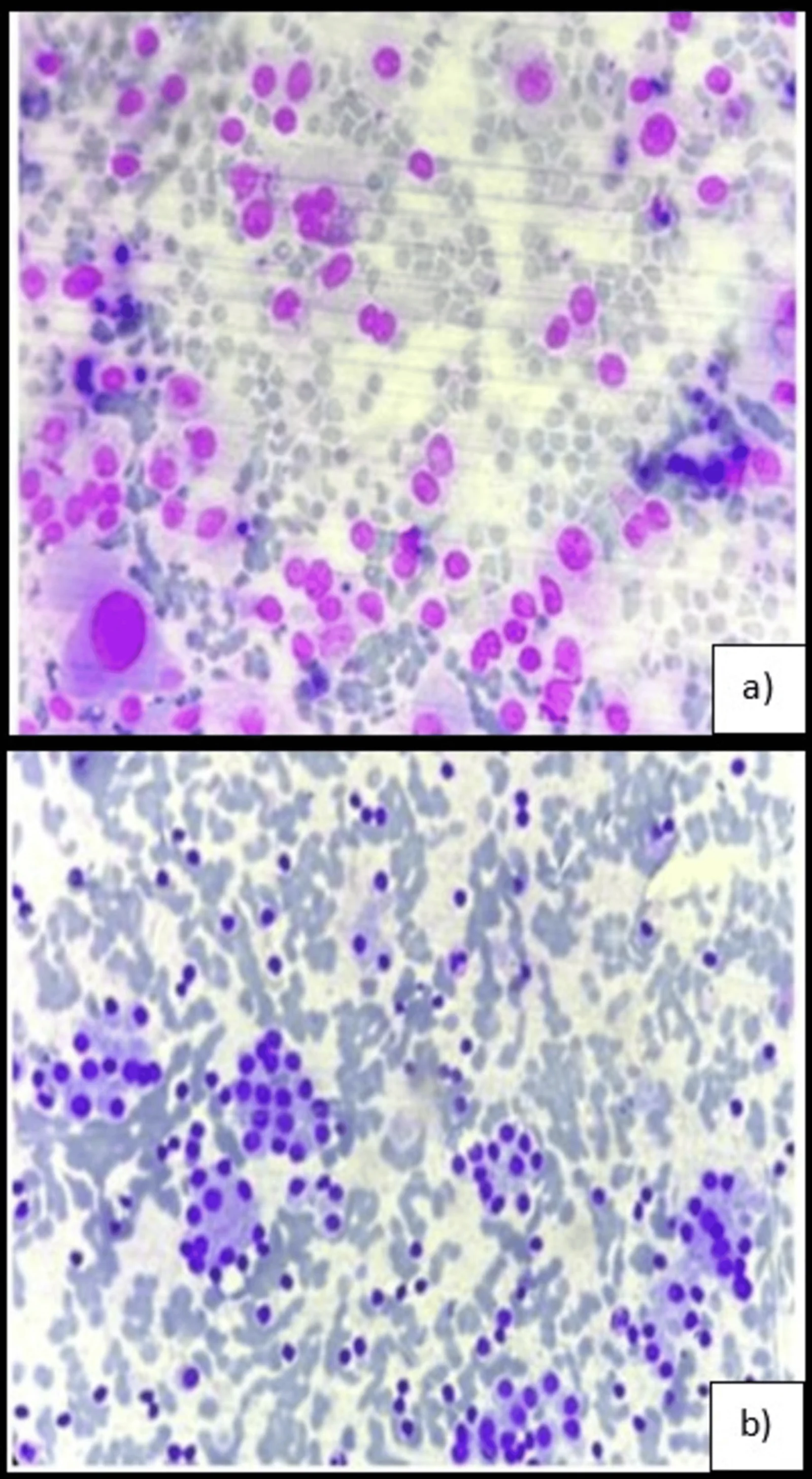
Atypia of undetermined significance (a) Atypia of undetermined significance -- nuclear atypia, rare cells show nuclear enlargement with slightly pale chromatin and irregular nuclear membrane (H&E stain, 400× magnification). (b) Atypia of undetermined significance -- other, architectural atypia showing follicular cells in focal microfollicular arrangement (MGG stain, 400× magnification). MGG, May-Grunwald-Giemsa; H&E, hematoxylin and eosin.

Nine cases were reported as Bethesda Category III (Atypia of Undetermined Significance (AUS)). Seven cytological smears were consistent with subcategory nuclear atypia; they showed few cells arranged in sheets and clusters with focal nuclear atypia (Figure 4a). Two cases showed occasional focal microfollicular arrangement of cells and were placed under the subcategory "other type" (Figure 4b).

**Figure 5:**
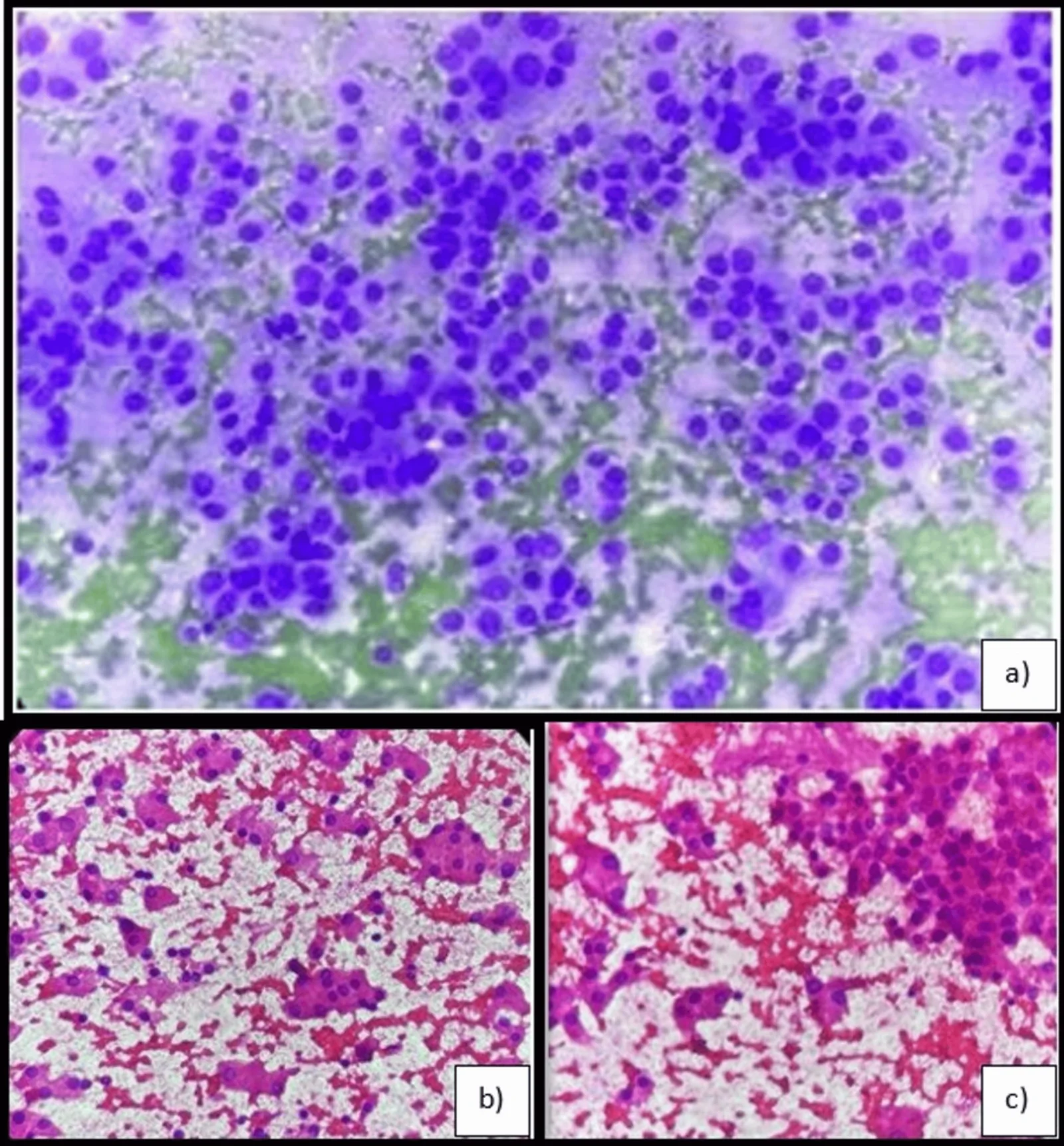
Follicular neoplasm (a) Follicular neoplasm, having monolayered fragments showing microfollicular arrangement of follicular cells (MGG stain, 400× magnification). (b, c) Oncocytic (Hurthle cell) follicular neoplasm, showing oncocytes with cytological atypia and enlarged nuclei (H&E stain, 400× magnification). MGG, May-Grunwald-Giemsa; H&E, hematoxylin and eosin.

Bethesda Category IV (Follicular Neoplasm) had eight cases; smears were moderately cellular, composed of thyroid follicular cells arranged in sheets and clusters, and formed microfollicles. Individual cells showed mild anisocytosis with enlarged nuclei and a scant to moderate amount of cytoplasm (Figure 5a). One case of oncocytic follicular neoplasm (OFN) showing large Hurthle cells with abundant cytoplasm and a centrally placed round nucleus was also reported (Figure 5b, 5c).

**Figure 6:**
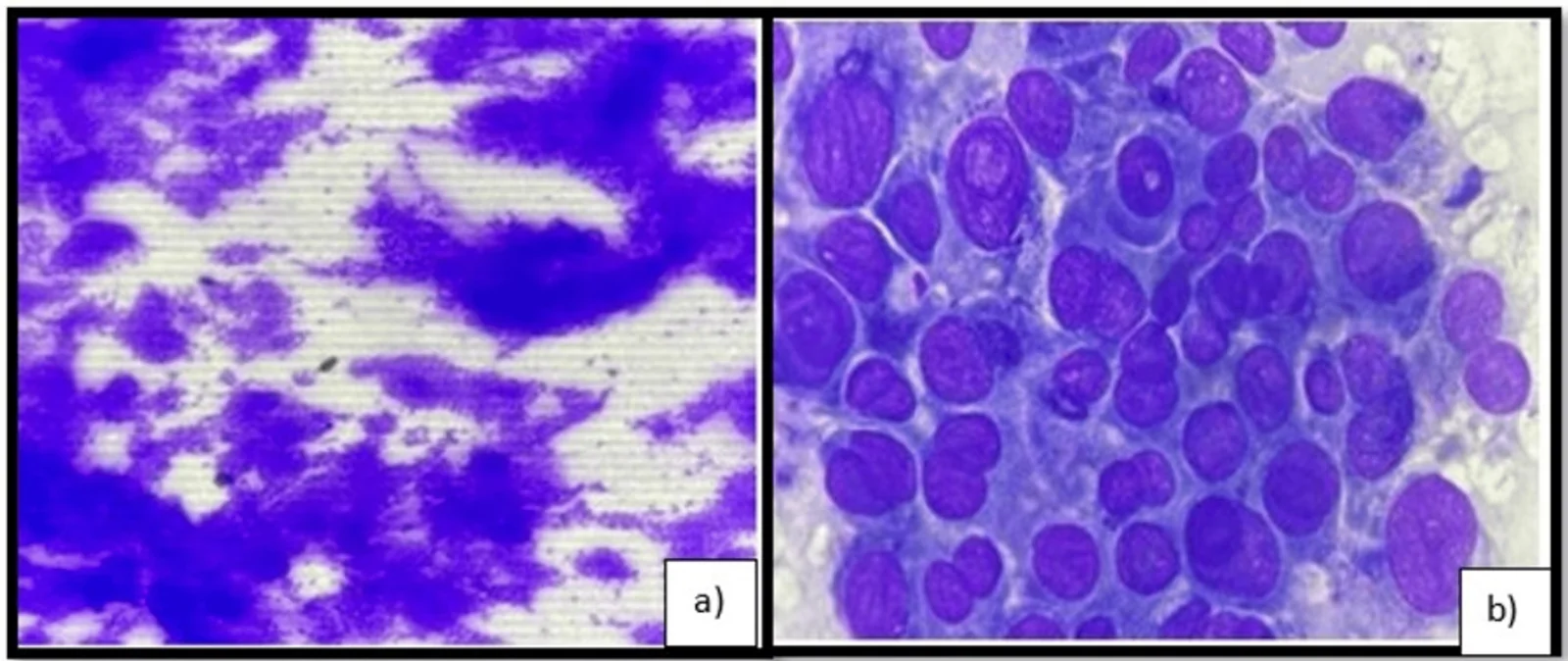
Malignant -- Papillary thyroid malignancy (a) Papillary thyroid carcinoma, having monolayered fragment showing papillary architecture (MGG stain, 100× magnification). (b) Papillary thyroid carcinoma, having monolayered flat sheets of cells with fine nuclear chromatin and intranuclear pseudoinclusions and nuclear grooves (MGG stain, 1000× magnification). MGG, May-Grunwald-Giemsa.

Bethesda Category V (Suspicious for Malignancy) consisted of eight cases. Smears were moderately cellular with cells showing moderate anisonucleosis, coarse chromatin, and prominent nucleoli (Figure 6a). Two cases showed binucleation, multinucleation, and intranuclear inclusions and were reported suspicious for papillary carcinoma (Figure 6b).

**Figure 7:**
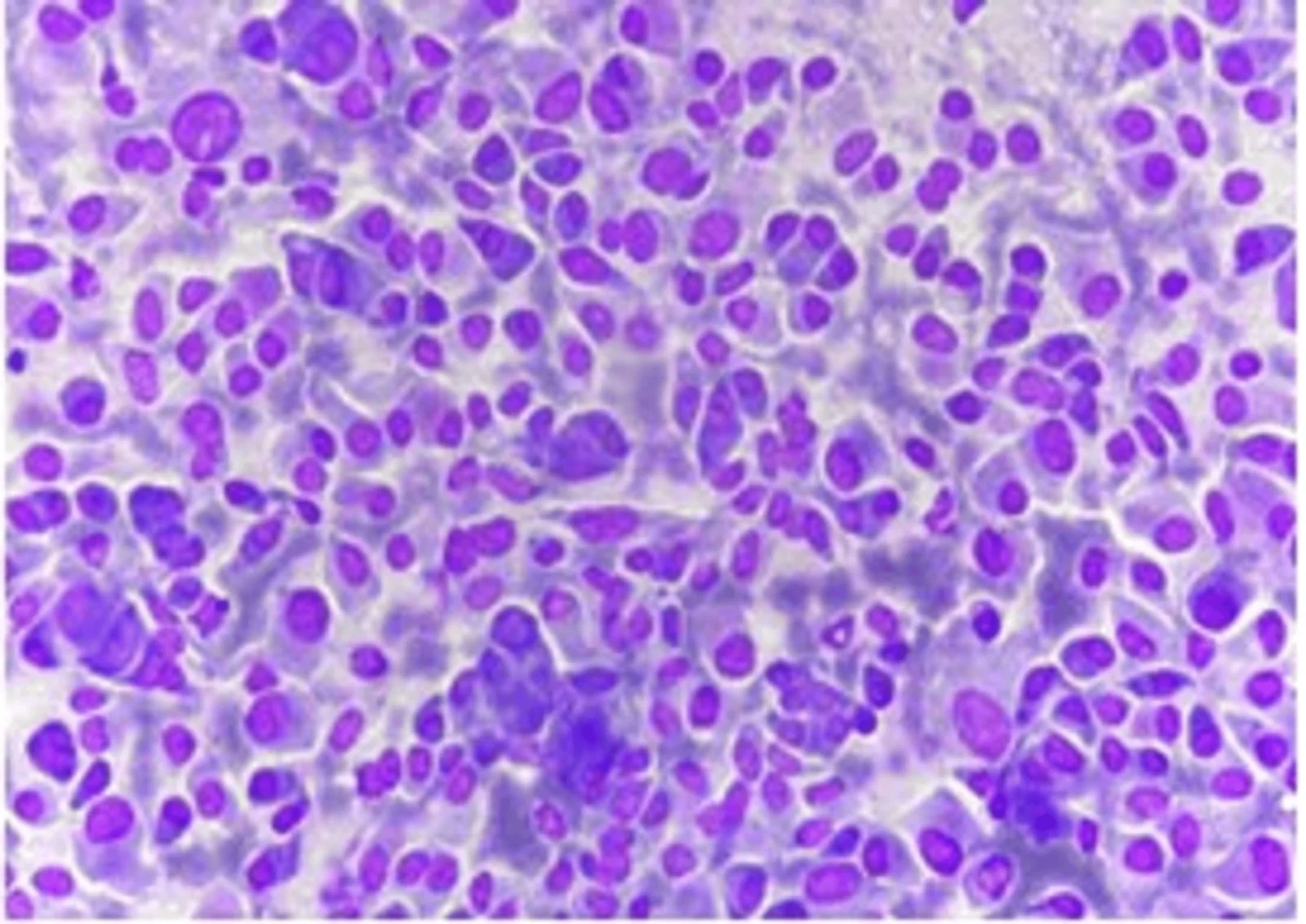
Malignant -- Medullary thyroid malignancy Medullary thyroid carcinoma, showing numerous polygonal, plasmacytoid cells and spindle cells in syncytial groups and as single cells, having salt-and-pepper chromatin (MGG stain, 400× magnification). MGG, May-Grunwald-Giemsa.

Only eight cases were reported as Bethesda Category VI (Malignant). In the malignant category, there were six cases of papillary thyroid carcinoma. These smears had abundant cellularity showing monolayered sheets of cells forming papillary architecture. Individual cells showed features of atypia, like increased nuclear-cytoplasmic ratio, irregular nuclear contour, fine chromatin, and prominent nucleoli. Focal areas showed nuclear overcrowding and nuclear overlapping. One case was reported as medullary thyroid carcinoma with the smear showing sheets and clusters of plasmacytoid cells and spindle cells (Figure 7).

**Figure 8:**
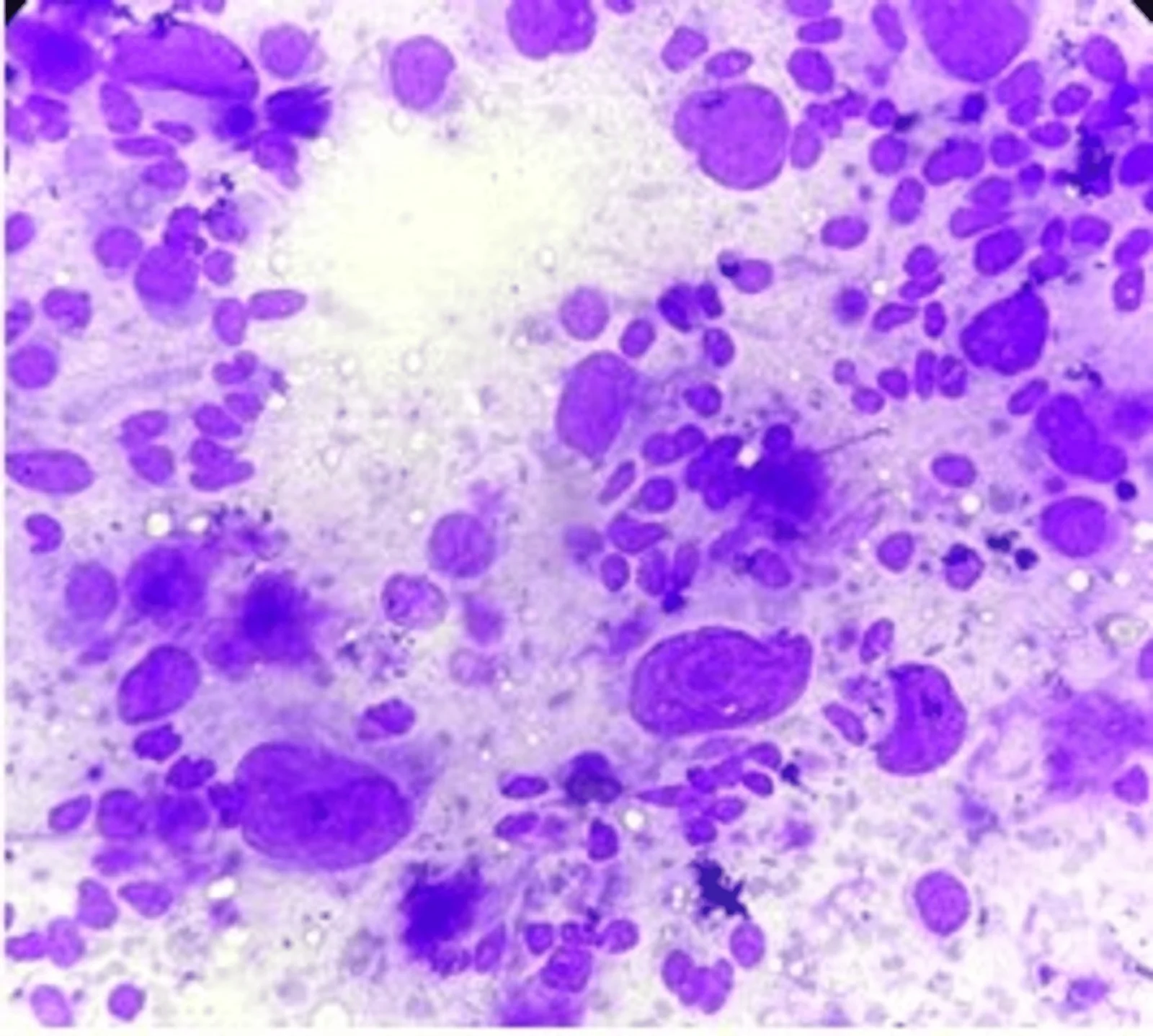
Malignant -- Undifferentiated (Anaplastic) thyroid malignancy Anaplastic thyroid carcinoma showing anaplastic tumor cells with marked nuclear pleomorphism and prominent nucleoli (MGG stain, 1000× magnification). MGG, May-Grunwald-Giemsa.

One smear was consistent with anaplastic thyroid malignancy, showing numerous spindle cells, large polygonal cells, and pleomorphic malignant cells with nuclear margins showing irregularity and prominent nucleoli (Figure 8). Multinucleated and bizarre cells were also noted.

Histopathological correlation

Surgical follow-up was available in 54 cases. There was good concordance between cytological and histopathological diagnoses, particularly in higher Bethesda categories (Table 6).

**Table 6:** Correlation of cytological diagnosis as per Bethesda system and histopathological outcome ROM, risk of malignancy.

Bethesda category	Total cases	Benign lesions	Malignant lesions	ROM	p-Value
I	0	0 (0.00%)	0 (0.00%)	0.00	--
II	39	39 (100.00%)	0 (0.00%)	0.00	<0.001
III	2	2 (100.00%)	0 (0.00%)	0.00	0.500
IV	6	5 (83.33%)	1 (16.67%)	16.67	0.250
V	3	0 (0.00%)	3 (100.00%)	100.00	<0.001
VI	4	0 (0.00%)	4 (100.00%)	100.00	<0.001
Total	54	46 (85.19%)	8 (14.81%)	14.81	--

Overall diagnostic performance

Accuracy was 100%, with perfect agreement between cytology (Bethesda V/VI as positive) and histopathology. Sensitivity and specificity were both 100%, indicating that all malignant cases were correctly identified (no false negatives) and all benign cases were correctly ruled out (no false positives). PPV and NPV were also 100%, reinforcing the reliability of Bethesda V and VI categories in predicting malignancy and Bethesda II in ruling it out. McNemar’s test (p = 1.000) confirms perfect diagnostic symmetry (Table 7).

**Table 7:** Diagnostic performance of cytology (Bethesda V/VI = positive) McNemar’s test for symmetry: p = 1.000 (perfect agreement).

Metric	Value (%)	95% CI	p-Value
Sensitivity	100.00	63.06-100.00	<0.001
Specificity	100.00	92.13-100.00	<0.001
Positive predictive value	100.00	59.04-100.00	<0.001
Negative predictive value	100.00	92.29-100.00	<0.001
Accuracy	100.00	93.40-100.00	<0.001

Histopathological profile of malignancies

Total malignancies: 8/54 (14.81%). Papillary carcinoma was the most common, 6 (75%), followed by follicular carcinoma 1 (12.5%) and medullary carcinoma 1 (12.5%). All malignancies in Bethesda V/VI were correctly identified, with no false positives or negatives (Table 8).

**Table 8:** Distribution of malignant histological subtypes

Malignant subtype	Number of cases (n = 8)	Percentage	p-Value
Papillary carcinoma	6	75	0.031
Follicular carcinoma	1	12	0.500
Medullary carcinoma	1	12.50	0.500
Total	8	100	--

Discordant case insight

The single malignant case within Bethesda category IV (follicular carcinoma) is consistent with the category's inherent, published ROM of approximately 30% (range 23-34%) as per TBSRTC 2023, rather than representing a true cytohistological discordance. Category IV is, by design, an indeterminate category: FNAC cannot distinguish follicular adenoma from follicular carcinoma, as this distinction requires histopathological demonstration of capsular and/or vascular invasion, which is not assessable on cytology.

## Discussion

Thyroid swellings are commonly encountered in endemic areas and clinically present either as a solitary nodule or as diffuse enlargement of the gland. Preoperative diagnosis is critical to avoid unnecessary surgeries in benign cases and to identify cases with malignant potential. FNA has been proven to be a safe and reliable diagnostic test. The Bethesda system provides a standardized platform for reporting thyroid FNAs, which has been helpful to clinicians in appropriate management.

Demographic profile

In the present study, over half of the cases (51.83%) were seen in the third and fourth decades of life, concordant with Singh et al. [9] and Barman and Banerjee [10], while Agarwal et al. [11] and Reddy et al. [12] reported a maximum number of cases slightly earlier, in the 11-30-year age group. This contrasts with Yassa et al.'s American cohort, which showed peak incidence between 40 and 50 years [13] -- a discrepancy plausibly attributable to earlier presentation of thyroid disease in Indian populations, related to a higher prevalence of nutritional deficiency and lower socioeconomic status. A strong female predominance was observed (male:female ratio 1:5.8), consistent with Barman and Banerjee [10], Yassa et al. [13], and Silverman et al. [14] (1:7, 1:7, and 1:10, respectively), reflecting the higher prevalence of autoimmune thyroid disease in female patients.

Bethesda category distribution

The overall category distribution in this study was broadly consistent with existing literature. The benign (Category II) proportion of 82.26% fell within the range reported by Barman and Banerjee [10], Agarwal et al. [11], Reddy et al. [12], Yassa et al. [13], and Guhamallick et al. [15] (60-89.2%), with Singh et al. [9] representing a notable outlier at only 57.3% benign cases, attributed to a comparatively higher proportion of suspicious/malignant reporting in that cohort. Within Category II, the proportion of follicular nodular disease (69.30%) and lymphocytic thyroiditis (10.42%) mirrored findings from Barman and Banerjee [10], Yassa et al. [13], Wu et al. [16], and Mondal et al. [17].

The non-diagnostic (Category I) rate of 8.45% closely matched Yassa et al. (7%) [13], though it was considerably lower than the 28.9% reported by Williams et al. [18], likely reflecting differences in sampling technique or adequacy criteria between centers. Category IV (Follicular Neoplasm) accounted for 2.25% of cases, concordant with Mondal et al. [17], Williams et al. [18], and Mufti and Molah [19] (4-4.4%) and lower than Agarwal et al. [11] and Yang et al. [20] (10.3% and 11.6%). Category V and VI proportions (2.25% each) were similarly comparable to Mondal et al. [17] and Williams et al. [18], with Yassa et al. [13] and Arul et al. [21] reporting somewhat higher rates for Category V, and Mufti and Molah [19] reporting a comparable Category VI rate against a higher figure from Yang et al. [20].

Diagnostic performance and histopathological correlation

Histopathological correlation was available in 54 cases. Diagnostic performance metrics (Bethesda V/VI as the positive test, Categories III/IV excluded as indeterminate) showed sensitivity, specificity, PPV, NPV, and accuracy of 100%, comparable to the 83.3-100% range reported by Singh et al. [9], Guhamallick et al. [15], and Mondal et al. [17]; the narrower discrepancy in the present study reflects the smaller subset to which these parameters were applied, relative to the full study sample.

The single malignant outcome within Category IV (follicular carcinoma, one of six surgically correlated cases) is consistent with the category's inherent, published ROM of approximately 30% (range 23-34%) per TBSRTC 2023, rather than representing a true cytohistological discordance [22]. Category IV is, by design, an indeterminate category: FNAC cannot reliably distinguish follicular adenoma from follicular carcinoma, as this distinction requires histopathological demonstration of capsular and/or vascular invasion, which is not assessable on cytology alone.

Diagnostic challenges in indeterminate categories

Categories III (AUS) and IV (Follicular Neoplasm) remain the most diagnostically challenging groups within TBSRTC, owing to overlapping cytomorphological features between reactive/benign atypia and true neoplasia. In this study, Category III accounted for 2.54% of cases (9/355), within the range generally reported in the literature. The wide published ROM range for both categories (13-30% for AUS; 23-34% for follicular neoplasm) reflects this diagnostic uncertainty and interobserver variability in applying the atypia threshold [22,23]. Ancillary strategies such as molecular testing and repeat FNA are increasingly used to refine risk stratification in these categories before surgical decision-making, though such testing was not performed in the present cohort.

Limitations

FNAC is a preliminary diagnostic test; immunohistochemical studies on cell blocks prepared from aspirates in Categories V and VI can provide a more accurate diagnosis. Histopathological correlation was available only in surgically managed cases, introducing a potential selection bias toward clinically suspicious lesions. The observed 100% diagnostic accuracy may be influenced by the relatively small number of cases with histopathological follow-up (n=54) and by the exclusion of indeterminate categories from this calculation, which could limit generalizability. Additionally, this was a retrospective, single-center study, which may limit the generalizability of findings to other populations and practice settings. Formal assessment of interobserver variability in Bethesda categorization was not performed, which represents a further limitation of the reporting methodology.

## Conclusions

FNA is a non-invasive, simple, reliable, and cost-effective preoperative procedure in patients presenting with thyroid swellings in order to triage patients and determine cases that require surgery. Along with biochemical and clinico-radiological findings, cytomorphological findings on FNA and reporting based on the Bethesda system proved to be useful in routine management. Implementation of TBSRTC reporting helps in reproducing reliable diagnoses among cytopathologists and also preserves the accuracy of reporting that helps and guides clinicians for definitive management, reducing the overall surgery rates and improving patient well-being. However, the near-perfect diagnostic performance observed in this study should be interpreted with caution, given the retrospective, single-center design, the relatively small number of histopathologically correlated cases, and the potential selection bias toward clinically suspicious lesions referred for surgery.

## References

[1] Tabaqchali MA, Hanson JM, Johnson SJ, Wadehra V, Lennard TW, Proud G (2000). Thyroid aspiration cytology in Newcastle: A six year cytology/histology correlation study. Ann R Coll Surg Engl.

[2] Chaudhari S, Hatwal D, Bhat P, Batra N, Bhat S (2015). Cytological evaluation of thyroid lesions and its correlation with histopathology: A prospective study. Int J Sci Stud.

[3] Amrikachi M, Ramzy I, Rubenfeld S, Wheeler TM (2001). Accuracy of fine-needle aspiration of thyroid. Arch Pathol Lab Med.

[4] Cibas ES, Ali SZ (2017). The 2017 Bethesda System for Reporting Thyroid Cytopathology. Thyroid.

[5] Ali SZ, Baloch ZW, Cochand-Priollet B, Schmitt FC, Vielh P, VanderLaan PA (2023). The 2023 Bethesda System for Reporting Thyroid Cytopathology. Thyroid.

[6] VanderLaan PA, Ali SZ (2023). Brief overview of the updated third edition of the Bethesda System for Reporting Thyroid Cytopathology. Diagn Histopathol.

[7] Zajdela A, de Maublanc MA, Schlienger P, Haye C (1986). Cytologic diagnosis of orbital and periorbital palpable tumors using fine-needle sampling without aspiration. Diagn Cytopathol.

[8] Nguyen GK (1991). Fine needle aspiration biopsy cytology of the thyroid: Its value and limitations in the diagnosis and management of solitary thyroid nodules. Pathol Annu.

[9] Singh DK, Shrivas RK, Paricharak DG, Nigam N, Nigam SK (2013). Role of fine needle aspiration cytology in solitary thyroid nodules. J Evol Med Dent Sci.

[10] Barman DD, Banerjee A (2018). Study of cytomorphological spectrum of various thyroid lesions and reporting under Bethesda system in tertiary care centre. J Med Sci Clin Res.

[11] Agarwal P, Dahiya S, Shams A (2019). Cytomorphological spectrum of thyroid lesions using Bethesda system of reporting with histopathological correlation: A study of 87 cases. Indian J Pathol Oncol.

[12] Reddy P, Prakash A, Giriyan SS (2018). Evaluation of Bethesda system for reporting thyroid cytology with histopathological correlation. Int J Res Med Sci.

[13] Yassa L, Cibas ES, Benson CB (2007). Long-term assessment of a multidisciplinary approach to thyroid nodule diagnostic evaluation. Cancer.

[14] Silverman JF, West RL, Larkin EW, Park HK, Finley JL, Swanson MS, Fore WW (1986). The role of fine-needle aspiration biopsy in the rapid diagnosis and management of thyroid neoplasm. Cancer.

[15] Guhamallick M, Sengupta S, Bhattacharya NK, Basu N, Roy S, Ghosh AK, Chowdhury M (2008). Cytodiagnosis of thyroid lesions-Usefulness and pitfalls: A study of 288 cases. J Cytol.

[16] Wu HH, Rose C, Elsheikh TM (2012). The Bethesda system for reporting thyroid cytopathology: An experience of 1,382 cases in a community practice setting with the implication for risk of neoplasm and risk of malignancy. Diagn Cytopathol.

[17] Mondal SK, Sinha S, Basak B, Roy DN, Sinha SK (2013). The Bethesda system for reporting thyroid fine needle aspirates: A cytologic study with histologic follow-up. J Cytol.

[18] Williams BA, Bullock MJ, Trites JR, Taylor SM, Hart RD (2013). Rates of thyroid malignancy by FNA diagnostic category. J Otolaryngol Head Neck Surg.

[19] Mufti ST, Molah R (2012). The Bethesda system for reporting thyroid cytopathology: A five-year retrospective review of one center experience. Int J Health Sci (Qassim).

[20] Yang J, Schnadig V, Logrono R, Wasserman PG (2007). Fine-needle aspiration of thyroid nodules: A study of 4703 patients with histologic and clinical correlations. Cancer.

[21] Arul P, Akshatha C, Masilamani S (2015). A study of malignancy rates in different diagnostic categories of the Bethesda system for reporting thyroid cytopathology: An institutional experience. Biomed J.

[22] Alshaikh S, Harb Z, Aljufairi E, Almahari SA (2018). Classification of thyroid fine-needle aspiration cytology into Bethesda categories: An institutional experience and review of the literature. Cytojournal.

[23] Bagıs M, Can N, Sut N (2024). A comprehensive approach to the thyroid Bethesda Category III (AUS) in the transition zone between 2nd edition and 3rd edition of the Bethesda System for Reporting Thyroid Cytopathology: Subcategorization, nuclear scoring, and more. Endocr Pathol.

